# Dorsal Striatal Circuits for Habits, Compulsions and Addictions

**DOI:** 10.3389/fnsys.2019.00028

**Published:** 2019-07-18

**Authors:** David M. Lipton, Ben J. Gonzales, Ami Citri

**Affiliations:** ^1^Edmond and Lily Safra Center for Brain Sciences, Hebrew University of Jerusalem, Jerusalem, Israel; ^2^Zuckerman Postdoctoral Scholar, Jerusalem, Israel; ^3^Institute of Life Sciences, Edmond J. Safra Campus, Hebrew University of Jerusalem, Jerusalem, Israel; ^4^Program in Child and Brain Development, MaRS Centre, Canadian Institute for Advanced Research, Toronto, ON, Canada

**Keywords:** habits, goal-directed behavior, striatum, prefrontal cortex, dorsomedial striatum, dorsolateral striatum

## Abstract

Here, we review the neural circuit bases of habits, compulsions, and addictions, behaviors which are all characterized by relatively automatic action performance. We discuss relevant studies, primarily from the rodent literature, and describe how major headway has been made in identifying the brain regions and neural cell types whose activity is modulated during the acquisition and performance of these automated behaviors. The dorsal striatum and cortical inputs to this structure have emerged as key players in the wider basal ganglia circuitry encoding behavioral automaticity, and changes in the activity of different neuronal cell-types in these brain regions have been shown to co-occur with the formation of automatic behaviors. We highlight how disordered functioning of these neural circuits can result in neuropsychiatric disorders, such as obsessive-compulsive disorder (OCD) and drug addiction. Finally, we discuss how the next phase of research in the field may benefit from integration of approaches for access to cells based on their genetic makeup, activity, connectivity and precise anatomical location.

## Bundles of Habits

“*When we look at living creatures from an outward point of view, one of the first things that strike us is that they are bundles of habits*” (James, 1890). Behavioral automaticity, as eloquently expressed in William James’ treatise “Habit,” is a fundamental aspect of our existence, and is essential for freeing-up our cognitive capacities so they can be directed to engaging novel and complex experiences, as further elaborated by James: “*The more of the details of our daily life we can hand over to the effortless custody of automatism, the more our higher powers of mind will be set free for their own proper work*.” (James, 1890). However, James also was very clear that these very same attributes of habits are also responsible for the most severe restrictions on our liberty. “*Habit is thus the enormous fly-wheel of society, its most precious conservative agent. It alone is what keeps us all within the bounds of ordinance…*” The topic of habit formation and its role in adaptive and maladaptive behavior has been extensively reviewed, most comprehensively in a recent dedicated issue of Current Opinion in Behavioral Science (Knowlton and Diedrichsen, 2018). Here, we provide a concise synthesis of the literature on the neural circuit basis of habits and their more extreme counterparts, compulsions and addictions, focusing on striatal circuits, which have primarily been deciphered in rodents. We begin with an overview of the common circuitry utilized by automatic behaviors, highlighting the importance of the dorsal striatum and inputs to this structure. We subsequently describe behavioral models used to study habits, compulsions and addictions, and then examine the neural circuit bases of these behaviors at increasingly higher resolution of analysis. We illustrate the established roles of the dorsolateral and dorsomedial subregions of the striatum in behavioral automaticity, and then review the complex picture of the roles of different striatal input structures, as well as specific cellular and synaptic modifications. Finally, we propose a roadmap for future investigations, integrating emerging molecular and circuit analysis methodologies with increasingly detailed knowledge of the multidimensional diversity of striatal cell-types, in order to analyze the circuits underlying automatic behaviors.

## What Are Habits, Compulsions, and Addictions and How Are They Related?

**Figure 1:**
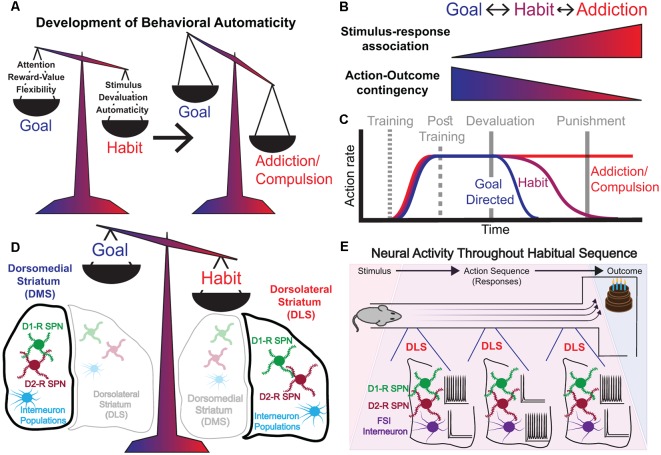
Characteristics of the shift from goal-directed to habitual behavior. **(A)** Left: Goal-directed and habitual behaviors are competitive processes that act in balance. Goal-directed behavior is characterized by a high requirement for attention, is highly contingent on present reward value, and demonstrates flexibility of responding. Habitual behavior is stimulus-driven, less dependent on present reward value, and governed by behavioral automaticity. Right: Addiction/compulsion represents an extreme state of habit. **(B)** The transition from goal-directed behavior to habitual behavior and then into compulsion, or addiction is graded. Shift from goal-directed to habitual behavior and then to compulsion/addiction corresponds to strengthened stimulus-response association and reduced action-outcome contingency. These processes are bidirectional, i.e., a behavior can shift on the spectrum from goal-directed to habitual performance, and back again—though in the extremes of addiction whether it is possible to return fully to habit/goal-directed states is less clear. **(C)** During instrumental training, rates of responding for a reward increase. Post-training reward devaluation reduces response rates more quickly for goal-directed behaviors than it does for habitual behaviors, which take many more extinction trials to fully dissipate. The extremes of addiction are characterized by compulsive responding that is resistant even to punishment. **(D)** The balance between goal-directed and habitual behavioral states corresponds to relative levels of neural activity in the dorsomedial (DMS) vs. dorsolateral (DLS) striatum. **(E)** Task-bracketing activity pattern emerges in the DLS as animals are over-trained on a rewarded behavioral sequence (e.g., running a T-maze for a tasty reward). Spiny Projection Neurons (SPNs) exhibit high activity at the beginning of a learned motor sequence and again at the end as the animal approaches the reward. Fast-spiking interneurons (FSIs) exhibit high activity during the middle stages of a behavioral sequence.

We intuitively use the term habit to describe behaviors that have become so ingrained that we perform them almost automatically, autonomously of the outcome (James, 1890; Dickinson, 1985; Graybiel, 2008; Robbins and Costa, 2017), and which, in extreme form, can become a compulsion or addiction. This is in contrast to goal-directed, purposeful behavior, in which an action is explicitly performed with the objective of obtaining a desired outcome (Valentin et al., 2007; Graybiel, 2008; Gremel and Costa, 2013; Robbins and Costa, 2017; Nonomura et al., 2018; Figures 1A,B). Goal-directed and habitual behaviors can be distinguished by their differential sensitivity to reward devaluation (i.e., reducing the value of the outcome; Figure 1C). Purposeful behavior will diminish if the outcome is no longer desired, while habitual performance will persist, since during the development of habitual behavior, the action becomes dissociated from the outcome, and performance is driven instead by antecedent stimuli and/or emotional states. Habitual behavior is therefore associated with behavioral automaticity, with diminished reliance on reinforcement. Thus, habits are shaped by past experience, and are characterized by computational efficiency and inflexibility, in contrast to goal-directed behavior, which is characterized by active deliberation of future consequences, high computational cost, and an adaptive flexibility to changing environments (Daw et al., 2005). Major benefits come from automaticity and independence from reinforcement, which allows the brain to free up rate-limiting attentional and decision-making resources. However, automaticity can also be detrimental, underlying the susceptibility to the development of maladaptive habits, which in the extreme can result in compulsions and addictions (Figures 1A,B). The central characteristic of compulsions and addictions is the continued pursuit of a previously rewarding stimulus, despite its clear current association with adverse consequences (Lüscher and Malenka, 2011; Volkow and Morales, 2015). This hallmark of addiction, action performance in spite of punishment, can be viewed as an extreme of habitual behavior (Figures 1A–C).

The intimate relationship of habits, compulsions and addictions is further made obvious by the coincident expression of behaviors of these categories. For instance, patients with obsessive-compulsive disorder (OCD) also demonstrate an enhanced tendency for dominance of habitual behavior (Gillan et al., 2011, 2016). Additionally, exposure to drugs of abuse, as well as binge-eating of palatable foods, enhance habit formation (Everitt and Robbins, 2016). Thus, cocaine addicts exhibit a higher tendency to form habits (Ersche et al., 2016), and alcohol exposure accelerates the emergence of habitual behavior (Corbit et al., 2012; Hogarth et al., 2012). These pathological states of behavioral automaticity have been shown to utilize overlapping circuitry.

## Common Limbic Circuitry Underlying Reinforcement Learning and Behavioral Automaticity

The neural circuits involved in instrumental learning and the automation of behavior (habits, compulsions, and addictions) include the striatum, midbrain dopaminergic nuclei, and regions of cortex that project to the striatum. These circuits are the primary focus of this review article, although it should be noted that the amygdala, thalamus, pallidum, and other limbic regions that are part of the broader basal ganglia circuitry are also involved in these behaviors. It has long been known that the striatum and its associated circuitry play a pivotal role in reinforcement learning and the development of behavioral automaticity found in habits, compulsions and addictions. The circuit composed of the ventral tegmental area (VTA) midbrain neurons projecting to the ventral striatum is considered to be the main circuit mediating reward and reward prediction error in the brain. Drugs of abuse target this circuit by either directly (e.g., nicotine) or indirectly (e.g., opioids) increasing midbrain dopamine neuron activity, and therefore enhancing dopamine signaling at release sites in the ventral striatum, or by directly inhibiting dopamine’s reuptake upon its release (e.g., cocaine; Lüscher, 2016). Thus, many studies of drug addiction have focused on neuroplastic changes that are induced in the ventral striatum following consumption of drugs of abuse (Lüscher and Malenka, 2011; Wolf, 2016). At the same time, habit formation has mostly been studied in the context of changes that occur in the dorsal striatum, which receives dopaminergic input from the Substantia Nigra Pars Compacta (SNc), while genetic mouse models of compulsion have focused on abnormal corticostriatal circuitry, largely involving dorsal striatum (Graybiel and Grafton, 2015; Smith and Graybiel, 2016). Thus, there has historically been a divided focus within the striatum, with ventral-striatal circuitry primarily investigated in the context of drug addiction, and dorsal-striatal circuitry in goal-directed and habitual reinforcement learning.

Over a decade ago, it was proposed that all of these instrumental behaviors ranging from habits to compulsions/addictions involve a shift in activity from the ventral to the dorsal striatum as habit learning progresses, and from the dorsomedial striatum to dorsolateral striatum as behavioral automaticity becomes more ingrained (Everitt and Robbins, 2005, 2013, 2016; Graybiel, 2008). The anatomy of corticostriatal circuits is well-suited to support such a mechanism, as the striatum is composed of spiraling loops through dopaminergic-striatal circuitry, ascending from the ventromedial to dorsolateral striatum (Haber et al., 2000; Haber, 2016). Here, we review the evidence that habits, compulsions and addictions are linked not only by their phenotype of behavioral automaticity but also by the underlying neural circuitry and plasticity mechanisms that give rise to them. This review article will focus on the essential role of dorsal-striatal circuits in encoding behavioral automaticity in several of its diverse manifestations.

## Experimental Paradigms Used to Model Habits, Compulsions and Addictions

Two major experimental paradigms have dominated the rodent literature on habits: (a) over-training (Jog et al., 1999; Graybiel, 2008; Smith and Graybiel, 2014); and (b) random interval (RI) training (Dickinson, 1985; Hilário et al., 2007; Rossi and Yin, 2012; Robbins and Costa, 2017). In both paradigms, animals are trained on an instrumental learning task, in which they learn to perform an action in order to obtain a reward. In over-training, an association between the stimulus and action (i.e., response) is formed and strengthened over the course of many more trials than are necessary for learning the task. During this overtraining, the stimulus-response association overwhelms the initially stronger relationship between the rewarding outcome and the contingent action (Graybiel, 2008; Smith and Graybiel, 2014). The strength of the stimulus-response association vs. that of the response-outcome is measured as the persistence in learned action performance during extinction trials following devaluation of the reward (Dickinson, 1985; Rossi and Yin, 2012). Thus, the rate of action performance following devaluation is used as a metric to assess the degree to which animals have become habit-entrained. Experimentally, such reward devaluation is often achieved by satiating the subject on the reward or pairing the reward with an aversive stimulus.

Though over-training is intuitive and advantageous in the simplicity of the experimental paradigm and framework, it is noteworthy that by definition, overtraining requires experimental subjects to perform many more trials than control subjects. This discrepancy in trial number forces an imbalance in experience between subjects and controls that may complicate analysis of the neural signatures of habit formation. An alternative approach to experimentally weaken the contingency between action and reward is RI training (Dickinson, 1985; Rossi and Yin, 2012; Robbins and Costa, 2017). In RI training, animals are trained to perform a specific action for a reward, which becomes available when the animal first successfully performs the required action after a random time interval has elapsed since the presentation of the previous reward. This paradigm promotes persistent, habitual behavior, as it is difficult for the subject to develop a clear association between action and outcome. A commonly used reference paradigm for RI training is random ratio (RR) training (Rossi and Yin, 2012), in which the contingency between the action and reward is more direct. RR training largely promotes similar behavioral output to RI training (similar rate of actions), while retaining goal-directed behavior, sensitive to devaluation (Figure 1C). In both overtraining and RI/RR paradigms, the contingency between action and outcome, or reward, is impacted, producing goal-directed behavior when response-outcome contingency is high, or habitual behavior when response-outcome contingency is low and stimulus-response contingency is high.

Drug addiction is modeled in animals in two principal ways: the first is non-contingent administration, where drugs are given to animals without being dependent on the animal’s response. The second is contingent drug self-administration, where the drug is delivered in response to an operant behavior, such as pressing a lever (Wolf, 2016). While non-contingent cocaine administration is advantageous in the experimental control over the parameters of cocaine exposure, self-administration more closely approximates the human experience of drug seeking, where individuals seek out drug-associated stimuli and perform responses that previously led to drug consumption (Wolf, 2016). Similar to habit learning, in drug self-administration, compulsive drug seeking can be studied during extinction trials, which are imposed after performance has passed a predefined criterion. Furthermore, drug self-administration also enables investigation of the impact of prolonged drug abstinence, during which it has been found that the degree of craving for the drug increases, a phenomenon termed “incubation of craving” (Wolf, 2016).

Rodent models of compulsive behaviors are largely based on tracking the performance of repetitive, stereotyped and seemingly purposeless behaviors, such as compulsive grooming (Ahmari, 2016). Importantly, OCD-like behaviors can emerge spontaneously, without a clear antecedent stimulus (Ahmari, 2016). These behaviors are primarily observed to develop naturally in genetically mutant rodents, rather than be induced by repeated instrumental learning.

## The Dorsolateral Striatum Plays a Key Role in Habit Formation and the Development of Compulsions/Addictions

The dorsal striatum is classically segregated into a medial aspect, the dorso-medial striatum (DMS), and a lateral aspect, the dorso-lateral striatum (DLS), both of which receive substantial cortical inputs. While the sensorimotor DLS receives major inputs from somatosensory and motor-cortical regions, the associative DMS receives major inputs from associative frontal cortical areas, such as orbitofrontal cortex (OFC; Berendse et al., 1979, 1992; Hintiryan et al., 2016; Hunnicutt et al., 2016). Classic studies have shown that the DMS is associated with goal-directed actions (Yin and Knowlton, 2004; Yin et al., 2005; Yin and Knowlton, 2006), while the DLS is associated with habitual actions (Balleine and Dickinson, 1998; Yin et al., 2004; Yin and Knowlton, 2006; Graybiel, 2008; Amaya and Smith, 2018; Figure 1D). Thus, goal-directed behavior is maintained after lesions to DLS (Yin et al., 2004; Yin and Knowlton, 2004, 2006), even following extended training, while lesions to DMS result in an early emergence of habitual behavior (Yin et al., 2005; Yin and Knowlton, 2006). The DLS has long been implicated in the performance of action sequences (O’Hare et al., 2018), both innate sequences such as grooming (Aldridge and Berridge, 1998), as well as acquired skills like learning to balance on an accelerating rotarod (Yin et al., 2009). These lesion-based studies provide the conceptual scaffold for our current understanding of the roles of the DMS and DLS in regulating goal-directed and habitual behavior.

Subsequently, a series of several influential studies on the roles of DMS and DLS in habit formation used tetrodes to track the activity patterns of neurons in the dorsal striatum while rats over-trained on a specific learning task: running a T-maze to obtain a food reward (Figure 1E). This led to the observation of *task-bracketing* patterns of activity in the DLS, which emerged concurrently with the acquisition of habitual behavior. In *task-bracketing* activity, highly-active DLS neurons have been reported to fire at the initiation and termination of the behavioral routine, an activity pattern that becomes strengthened with over-training (Jog et al., 1999; Barnes et al., 2005; Thorn et al., 2010; Smith and Graybiel, 2013; Figure 1E). Importantly, such task-bracketing, or action-sequence related activity in the DLS has also been observed in rats (Martiros et al., 2018) and mice (Jin and Costa, 2010; Jin et al., 2014) during a sequential lever-pressing task. A contrasting phenomenon is observed in the DMS, where neural activity is elevated more consistently throughout the performance of a behavioral routine, especially during the initial phases of acquisition of a novel instrumental behavior (Yin et al., 2009; Thorn et al., 2010; Gremel and Costa, 2013). This DMS activity then subsides as animals become over-trained (Yin et al., 2009; Gremel and Costa, 2013), corresponding to the time frame when task-bracketing activity emerges in the DLS. It should be noted that the task bracketing activity in DLS was observed in a subset of the most highly active neurons in this sub-region (Barnes et al., 2005; Martiros et al., 2018). Indeed, the majority of neurons in the DLS exhibit activity throughout the execution of the entire habit routine: in mice that were well-trained to habitually accelerate running on a treadmill to obtain a reward, neural activity was engaged in the DLS throughout the routine, with different striatal neurons encoding different sensorimotor features of the task (Rueda-orozco and Robbe, 2015).

Notably, multiple sources of evidence suggest that DLS control of habitual behavior and DMS control of goal-directed behavior likely develop in parallel and can varyingly compete or cooperate for control over actions (Daw et al., 2005; Yin and Knowlton, 2006; Gremel and Costa, 2013; Smith and Graybiel, 2014; Kupferschmidt et al., 2017; Robbins and Costa, 2017). For instance, inactivation of the DLS after the establishment of habitual behavior can restore goal-directed responding (Yin and Knowlton, 2006). Furthermore, DLS lesions or optogenetic silencing can expedite learning early in training (Bradfield and Balleine, 2013; Bergstrom et al., 2018), possibly by shifting control to goal-directed systems. Thus, a key transition thought to occur during the formation of habits is the relative quieting of activity in DMS, coincident with generally elevated activity in DLS, including task-bracketing (Thorn et al., 2010; Gremel and Costa, 2013).

In compulsions, the dorsal striatum also plays a central role, as several studies of genetic models of OCD, notably the SAPAP3^−/−^ model, have indicated that activity in striatal circuits is disrupted coincident with the expression of compulsive behavior. As will be subsequently discussed, these studies focused on the striatal regions to which the orbitofrontal/secondary motor cortical areas project, encompassing the ventromedial (Ahmari et al., 2013), centromedial (Burguière et al., 2013), and central subregions of the dorsal striatum (Corbit et al., 2019). Additionally, there is evidence that the dorsolateral striatum is functionally necessary for the sequencing of compulsive grooming, as rats with lesions of the DLS express disruptions in the stereotypy of grooming sequences (Cromwell and Berridge, 1996; Kalueff et al., 2016).

In contrast to studies on habit formation and compulsions, centering mostly on the dorsal striatum, the majority of studies on drug addiction have focused on the mesolimbic, ventral striatal “reward” pathway (Lüscher and Malenka, 2011; Volkow and Morales, 2015; Wolf, 2016; Francis et al., 2019). Studies of the dorsal striatum that have addressed drug-seeking behavior (primarily in the study of alcohol and cocaine) have shown it to associate with a medial-lateral transition in neural activity in this subregion (Corbit, 2018). Prolonged cocaine self-administration in rats results in a persistence of cocaine seeking, even in the presence of active punishment (Vanderschuren and Everitt, 2004). During this cued cocaine self-administration, dopamine release is detected in the dorsal striatum (Ito et al., 2002), and inactivating the DLS blocks punishment-resistant seeking of drug-predicting cues (Jonkman et al., 2012). Indeed, while activity in ventral striatal circuits is clearly essential for the development of compulsive cocaine seeking, after prolonged administration, dorsal-striatal circuits become increasingly engaged, to support drug seeking (Belin and Everitt, 2008; Belin et al., 2009). Furthermore, once the dorsal striatum is engaged, there is a further activity shift, from DMS-centric to DLS-centric. Initially, drug seeking is goal-directed, and depends on a network involving the DMS (Corbit et al., 2012; Murray et al., 2014). However, after prolonged exposure, drug seeking becomes habitual, depending on neural activity and dopamine action in the DLS. Indeed, rats trained to press a lever for cocaine reward will reduce their lever pressing due to perfusion of dopamine receptor antagonists in DMS early in training and in DLS following over-training (Vanderschuren et al., 2005; Murray et al., 2012). This reduction in drug seeking was also observed in rats as a consequence of lidocaine-induced DLS inactivation (Zapata et al., 2010). Additionally, alcohol exposure has been reported to disinhibit Spiny Projection Neurons (SPNs) in the DLS, providing a potential mechanism for the transition to automaticity (Wilcox et al., 2014; Patton et al., 2016). In addition, the DLS has been shown to be necessary in rats for the development of habitual heroin seeking (Hodebourg et al., 2018). Furthermore, long-term exposure to nicotine alters synaptic plasticity in the DLS of rats, perturbing endocannabinoid-mediated long-term depression (LTD; Adermark et al., 2019). Thus, the dorsal striatum, and particularly the DLS, is implicated in the development of habitual drug-seeking. However, it should be emphasized that the amount of evidence on the role of the dorsal striatum in drug-addiction still lags behind what is known for the ventral striatum. Further research will help clarify the role of the dorsal striatum in addictive behaviors.

## Corticostriatal Circuitry and Other Limbic Circuits Underlying Behavioral Automaticity

The striatum receives inputs from multiple cortical regions (Webster, 1961; Beckstead, 1979; Hintiryan et al., 2016; Hunnicutt et al., 2016), and prefrontal inputs to the striatum have been shown to play significant roles in both goal-directed, as well as habitual behavior (Gourley and Taylor, 2016; Smith and Laiks, 2017; Amaya and Smith, 2018). The major frontal structures that have been implicated in instrumental and automatic behaviors are the prelimbic cortex (PL) and infralimbic cortex (IL) Amaya and Smith, 2018 in the medial prefrontal cortex (mPFC), as well as the OFC located in the ventral part of the PFC.

Interestingly, the two substructures of the mPFC, the IL and PL, seem to play opposing roles in balancing between goal and habit, with the IL supporting habitual behavior, and the PL supporting goal-directed behavior (Smith and Laiks, 2017; Amaya and Smith, 2018). The IL exhibits task-bracketing activity, similar to the activity observed in the DLS during habit learning (Smith and Graybiel, 2013). Furthermore, chronic perturbation of the IL disrupts both habit acquisition and expression (Smith et al., 2012; Smith and Graybiel, 2013), while its optogenetic inhibition disrupts habit expression (Smith et al., 2012).

Meanwhile, lesions to the PL of rats reduced their ability to act in a goal-directed manner, biasing the rats toward habitual behavior (Balleine and Dickinson, 1998; Corbit and Balleine, 2003; Killcross and Coutureau, 2003; Balleine and O’Doherty, 2010). Indeed, recent studies in rats have shown that PL inputs to the posterior DMS (pDMS) are necessary for goal-directed learning: in rats lacking this PL-pDMS connection, there is a failure to reduce instrumental responding after reward devaluation (Hart et al., 2018a,b). Thus, reducing the strength of the PL- input to the DMS might permit the development of automaticity, mediated through sensorimotor corticostriatal circuits converging on the DLS. Indeed, reduced activity of PL neurons was observed in rats that underwent extended training for cocaine self-administration; meanwhile, stimulating PL neurons reduced the extent of compulsive cocaine seeking in these compulsively self-administering rats (Chen et al., 2013). Together, these data make a strong case that activity in the IL is important for habitual behavior, while PL activity facilitates goal-directed behavior.

However, many reports complicate this simple IL = habit; PL = goal-directed view. For instance, the PL is reported to be involved in facilitating post-extinction reinstatement of drug-seeking. This reinstatement of drug-responding can be elicited by re-exposure to drug-associated cues, consumption of the drug itself, or a stressful experience (McFarland and Kalivas, 2001; McFarland et al., 2004; Gipson et al., 2013; Ma et al., 2014; Moorman et al., 2015; Gourley and Taylor, 2016; McGlinchey et al., 2016). At the same time, there is evidence supporting a role for the IL in driving drug-cue extinction learning (Peters et al., 2008; Ma et al., 2014; Moorman et al., 2015; Gourley and Taylor, 2016; Gutman et al., 2017), as opposed to habit-expression. Together, these results suggest that the PL, in general, mediates a “go” signal, driving drug-seeking responses, particularly during post-extinction reinstatement, whereas in contrast, the IL sends a “no-go” signal, necessary for extinction in drug-reward instrumental learning (Moorman et al., 2015; Gourley and Taylor, 2016). These results are potentially conflicting with the habit-literature, as IL promotes extinction of responding in the drug-reward paradigm, and seems to facilitate responding in habit learning paradigms, while PL also can play contrasting roles in each paradigm. One possible explanation for this discrepancy is that where specific projections from mPFC (PL and IL) to striatum are examined in drug-seeking, they are those to the ventral striatum (McFarland and Kalivas, 2001; Peters et al., 2008; Ma et al., 2014; Gourley and Taylor, 2016). Conversely, in habit formation, the projections from PL/IL to regions of dorsal striatum have been given more attention (Smith and Laiks, 2017; Hart et al., 2018a,b).

The OFC also plays an important role in instrumental behaviors, with evidence appearing to support the idea of the OFC promoting goal-directed behavior. However, the OFC is a large cortical structure, with multiple subregions, and its roles in instrumental behavior and economic choice appear to be varied and complex (Stalnaker et al., 2015; Gremel et al., 2016; Gardner et al., 2018; Panayi and Killcross, 2018; Zhou et al., 2019). The OFC receives multisensory input (Gourley and Taylor, 2016), projects to the anterior/intermediate DMS and central region of the striatum, and has been shown to exhibit activity that correlates with the reward assigned to a given stimulus (Zhou et al., 2019). The OFC exhibits greater activity during goal-directed behavior, and, similar to DMS neurons, is particularly active during random-ratio lever-pressing training, when action-reward contingency is high (Gremel and Costa, 2013; Gremel et al., 2016). OFC stimulation can increase the degree to which mice are goal-directed, and reduce the degree to which mice are habit-driven in lever-pressing (Gremel et al., 2016). Furthermore, endocannabinoid-dependent (eCB)-LTD of the OFC inputs to the DMS biases mice towards habitual behavior, providing further evidence for a competition between goal-directed and habitual behavior—such that if the activity of the OFC-DMS pathway is decreased (e.g., through eCB-LTD), then the DLS pathway prevails, promoting habitual behavior (Gremel et al., 2016).

Interestingly, OFC-striatal circuits are also implicated in compulsive behavioral automaticity. Abnormalities of the structure, connectivity and activity of the caudate (the human DMS) have been observed in OCD patients (Carmin et al., 2002; Guehl et al., 2008; Sakai et al., 2011; Fan et al., 2012). Furthermore, three genetic mouse models of OCD have been characterized (*D1CT-7*; *SAPAP3*^−/−^ and *Slitrk5*^−/−^), and in each of them, the major circuit phenotype observed has been disruption of cortico-striatal synaptic transmission, particularly involving inputs from OFC (Nordstrom and Burton, 2002; Welch et al., 2007; Shmelkov et al., 2010; Burguière et al., 2013, 2015). Indeed, chronic activation of the medial OFC leads to the development of OCD-like grooming behavior in mice, and drives sustained activity of ventromedial striatal SPNs (Ahmari et al., 2013). In contrast, optogenetic stimulation of the lateral OFC (lOFC) has been reported to reduce the occurrence of grooming behaviors in genetically modified mice that compulsively over-groom, while activating feed-forward inhibition within the striatum (Burguière et al., 2013). Furthermore, a recent report compared lateral OFC-striatal circuit activity to the activity in projections from neighboring M2 cortex, in the SAPAP3^−/−^ mouse model of OCD. They found that in the SAPAP3^−/−^ mutant, lOFC input to striatal SPNs was reduced in strength, while M2 input to both SPNs and fast-spiking interneurons (FSIs) in striatum was increased 6-fold, suggesting that it is M2, and not lOFC inputs, that drive compulsive grooming (Corbit et al., 2019). Meanwhile, another study found that compulsive consumption of ethanol resulted in reduced OFC input to D1R-expressing DMS neurons during ethanol withdrawal, reducing goal-directed behavior, and resulting in habitual alcohol consumption (Renteria et al., 2018). Thus, many of these recent results suggest that OFC hypoactivity corresponds with automatic behavior and at least in some cases, activating OFC projections can counteract this automaticity, rather than drive it. However, in another recent article describing a mouse model of addiction (based on self-stimulation of VTA-dopamine neurons), potentiation of synapses from the lOFC to the central part of dorsal striatum was observed (Pascoli et al., 2018). Thus, while there is significant literature documenting the involvement of OFC projections to striatum in behavioral automaticity, the OFC appears to play varied roles in either facilitating or countering automaticity. Therefore, further research is required in order to clarify the principles of OFC-striatal connections and their role in driving and/or inhibiting automatic behavior.

As another main input source to the striatum, midbrain dopamine neurons are an essential component of the reward circuitry, and such neurons in both the VTA and SNc send collaterals to the striatum, PFC, and other forebrain targets (Volkow and Morales, 2015; Everitt and Robbins, 2016; Lüscher, 2016). Dopamine is a crucial modulator of striatal action and the transition from goal-directed to habitual behavior (Graybiel, 2008; Everitt and Robbins, 2016). It is well established that the cellular activity of midbrain dopamine neurons is increased upon exposure to rewarding drugs, in large part due to the strengthening of synaptic inputs onto these dopamine neurons (Ungless et al., 2001; Lammel et al., 2011; Creed et al., 2016; Francis et al., 2019). Plasticity mechanisms are also engaged within midbrain dopamine neurons during the formation of a naturally rewarded (i.e., food-reward) habit, as habitual responding after devaluation on a random-interval lever-press habit depends on this population’s expression of NMDA receptors (Wang et al., 2011).

Finally, an additional striatum-associated structure that has been implicated in habitual and addictive behavior is the amygdala (Lingawi and Balleine, 2012). Conceptually, the amygdalar connection is intriguing, as habit formation is exacerbated by stress (Dias-Ferreira et al., 2009), in a process that may be mediated by amygdalar-striatal circuits. One recent study demonstrated that both the basolateral and central amygdala (BLA and CeA) exert control over habitual behavior in rats; the BLA was found to be involved in habitual responding early in training, with the CeA playing a crucial role in generating habitual responding later in extended training (Murray et al., 2015). These amygdalar circuits, and the BLA in particular, play a key role in assigning valence, and have been shown to play a role in appetitive behaviors (Kim et al., 2017) while the CeA has been shown to play a role in alcohol addiction (de Guglielmo et al., 2019). Neither nucleus has direct connections to the DLS (Murray et al., 2015; Hunnicutt et al., 2016), and therefore the amygdala likely influences the DLS through multisynaptic connections. Given the direct projection of BLA neurons to the ventral striatum, these amygdalar circuits could influence dorsal striatal circuitry *via* ventral striatum (Murray et al., 2015).

Overall, we have focused on the brain regions that represent key nodes in the circuitry of habitual and compulsive behavior. Eventually however, continued and disordered performance of instrumental behaviors, particularly as occurs in chronic drug use, leads to alterations in reward and attentional related networks that likely involve changes to additional brain structures, such as the ventral hippocampus, and insular cortex (Everitt and Robbins, 2016). Other key structures involved in broader basal ganglia circuits also likely play important roles in encoding behavioral automaticity. For instance, thalamus sends a significant projection to striatum (Hunnicutt et al., 2016), and specific projections from thalamic nuclei to the DMS are necessary for goal-oriented behavioral flexibility (Bradfield et al., 2013; Díaz-Hernández et al., 2018).

## Striatal Cell-Types, Microcircuits, and Their Specific Contributions to Habits and Compulsions

Within the striatum the vast majority of neurons (>90%) are SPNs, which are roughly evenly split between Dopamine D1 receptor (Drd1)-expressing direct pathway SPNs (dSPNs; projecting directly to the midbrain nucleus, Substantia Nigra reticulata, or SNr, as well as Globus Pallidus internus, or GPi) and Drd2-expressing indirect pathway SPNs (iSPNs; projecting to the Globus Pallidus externus, or GPe; Kreitzer and Malenka, 2008; Burke et al., 2017). The striatum also contains populations of interneurons, including Cholinergic (ChAT) and Parvalbumin-expressing Fast-Spiking Interneurons (PV+ FSIs) (Kreitzer and Malenka, 2008; Burke et al., 2017).

Over the past decade, progress has been made in deciphering the roles of dSPNs vs. iSPNs in motor behavior, action initiation, and reinforcement learning, all of which are combined to produce habitual and compulsive behaviors. A decade ago, a seminal study confirmed the prevalent assumption in the field that dSPNs in the direct pathway serve to promote actions/behaviors, while iSPNs in the indirect pathway inhibited behaviors (Kravitz et al., 2010; Bariselli et al., 2019). However, it is now apparent that dSPNs and iSPNs are concurrently activated during the initiation of actions (Cui et al., 2013; Tecuapetla et al., 2014, 2016), and thus the role of iSPNs seems to be more complex than simple broad behavioral inhibition (Tecuapetla et al., 2016; Vicente et al., 2016; Parker et al., 2018; Bariselli et al., 2019). Moreover, patterns of activity in locally concentrated clusters of both dSPNs and iSPNs have been recently observed to correspond to specific actions, like turning left or right (Barbera et al., 2016; Klaus et al., 2017; Markowitz et al., 2018; Parker et al., 2018). Still, several studies have found that dSPNs are activated with shorter latency than iSPNs during action initiation (Sippy et al., 2015; O’Hare et al., 2016). Meanwhile, other studies have demonstrated that dSPN activation reinforces the performance of specific action patterns (Sippy et al., 2015; Vicente et al., 2016), while iSPN activation might weakly reinforce actions more generally (Vicente et al., 2016) in some contexts, and inhibit action performance in others (Kravitz et al., 2010; Sippy et al., 2015). Thus, both dSPNs and iSPNs are likely to be engaged in both the learning and the execution of a habit, with dSPN activity likely to promote action performance, and iSPN activity likely to play an action-specific inhibitory and/or permissive role (Zalocusky et al., 2016; Parker et al., 2018; Bariselli et al., 2019). How exactly these SPN pathways coordinate and are modified during instrumental learning is currently still a topic of active research (Bariselli et al., 2019).

In addition to SPNs, recent studies in rodents have also implicated FSIs in the development of habits (Thorn and Graybiel, 2014; O’Hare et al., 2017; Martiros et al., 2018). For instance, FSIs are active during the middle phase of a lever-pressing motor sequence pattern, when the activity of *task-bracketing* SPNs is reduced (Martiros et al., 2018). In the context of compulsive behavior, in one of the OCD mouse models (*SAPAP3*^−/−^), a reduction in the number of striatal PV neurons was observed, leading to a reduction in feed-forward inhibition, potentially reducing inhibition of cortico-striatal inputs (Burguière et al., 2013). A reduction in striatal PV neurons has also been reported in patients suffering from Tourette’s syndrome (Kalanithi et al., 2005), a syndrome of ritualized, repetitive actions. Furthermore, selective ablation of striatal PV interneurons in mice has been reported to lead to increased stereotypic grooming, a measure of OCD-like behavior in rodents (Kalueff et al., 2016). In all of these examples, reduced activity of FSI interneurons leads to increased SPN activity, potentially leading to the promotion of automatic behaviors. In addition, striatal cholinergic interneurons also play a significant role in modulating SPN plasticity (Augustin et al., 2018), and are thought to mediate thalamic influence on striatal circuits involved in goal-directed behaviors (Bradfield et al., 2013; Peak et al., 2019).

## Synaptic and Molecular Changes in Limbic Circuits for Behavioral Automaticity

In the context of addiction, significant progress has been made in determining how drugs of abuse affect synaptic plasticity in the mesolimbic ventral-striatal reward system, involving the VTA and ventral striatum, or Nucleus Accumbens (NAc). These mechanisms are extensively summarized elsewhere (Citri and Malenka, 2008; Lüscher and Malenka, 2011; Lüscher, 2016; Wolf, 2016; Francis et al., 2019). Yet, in the context of this review, there are several important principles to emerge that are worth mentioning. First, synaptic plasticity mechanisms in both the VTA and NAc involve dopamine and NMDAR-receptor dependent long-term plasticity (Ungless et al., 2001; Saal et al., 2003; Conrad et al., 2008; Lüscher and Malenka, 2011; Wolf, 2016). Second, these changes are input-specific, occurring at particular synaptic inputs onto VTA or NAc neurons (Lammel et al., 2011; Ma et al., 2014; MacAskill et al., 2014; Pascoli et al., 2014; Wolf, 2016; Barrientos et al., 2018). Finally, plasticity following exposure to drugs of abuse is dynamically regulated (Thomas et al., 2001; Kourrich et al., 2007; Lüscher and Malenka, 2011; Wolf, 2016). These rules of cellular and synaptic plasticity in the VTA-NAc circuit could provide a useful template for how mechanisms of plasticity in DLS circuitry might proceed.

Focusing on the dorsal striatum and natural reward habits, synaptic modulation has been observed in accordance with behavioral automaticity, principally at corticostriatal synapses. Indeed, the acquisition of goal-directed actions has been associated with synaptic plasticity at corticostriatal synapses within the DMS, enhancing transmission onto dSPNs, while weakening inputs onto iSPNs (Shan et al., 2014). Meanwhile, in mouse brain slices of habit-entrained mice, it was observed that inputs onto both dSPNs and iSPNs in dorsal striatum were strengthened, though inputs to dSPNs were activated with a shorter latency and moreover, habit suppression correlated with reduced activity of only dSPNs (O’Hare et al., 2016). Furthermore, glutamatergic synapses from secondary motor cortex onto DLS dSPNs (and not iSPNs) were observed to be strengthened with learning of simple sequences (Rothwell et al., 2015). All these studies suggest a selective modification of corticostriatal-dSPN synapses. However, during the learning of a rotorod-balancing skill, it was found that synaptic strength onto iSPNs in the DLS strengthened with training and was crucial for acquisition of skilled balancing (Yin et al., 2009), and so corticostriatal-iSPN synapses are likely important as well. In the studies mentioned thus far, synaptic changes recorded were post-synaptic. Yet, one elegant study, also examining striatal inputs in mice during rotorod balancing, found learning-induced activity differences in somata vs. pre-synaptic terminals from mPFC and M1 corticostriatal neurons, suggesting neuroplastic changes that were specific to pre-synaptic terminals during learning (Kupferschmidt et al., 2017). In the context of compulsions, in the Sapap3 mutant mice, which exhibit increased grooming, reduced synaptic transmission of corticostriatal synapses onto dSPNs (but not iSPNs) was observed, as measured by mESPC frequency (Wan et al., 2013). This finding is consistent with much of the learned skill/habit literature. To summarize, synaptic changes have been observed to occur in dorsal striatum during the learning of both goal-directed and habitual behaviors, mostly strengthening inputs onto DMS and DLS neurons, respectively. Clearly though, much more research remains to be done to decipher how habits and compulsions result from the modification of cell-type specific synapses within striatum, e.g., inputs to dSPNs, iSPNs, and local interneurons in striatum.

## Facing Forward

In this review article, we have summarized the overlapping dorsal-striatal-centric circuitry responsible for learning habits, addictions, and compulsions, highlighting the transition from DMS to DLS as behaviors become more automatic. With this overarching framework in mind, we examine future directions concerning the mechanisms of behavioral automaticity and propose how our current understanding of different features of striatal circuit organization can be combined with novel molecular tools to provide insight into the central questions in the field. One crucial question is how dispersed is the representation of a given automatic behavior within the dorsal striatum? If the shift to automaticity involves the transition from DMS- to DLS-centric circuits, then is the same S-R behavior encoded simultaneously in medial and lateral locations, and furthermore what particular cells and synapses correspond to the storage of a given association?

**Figure 2:**
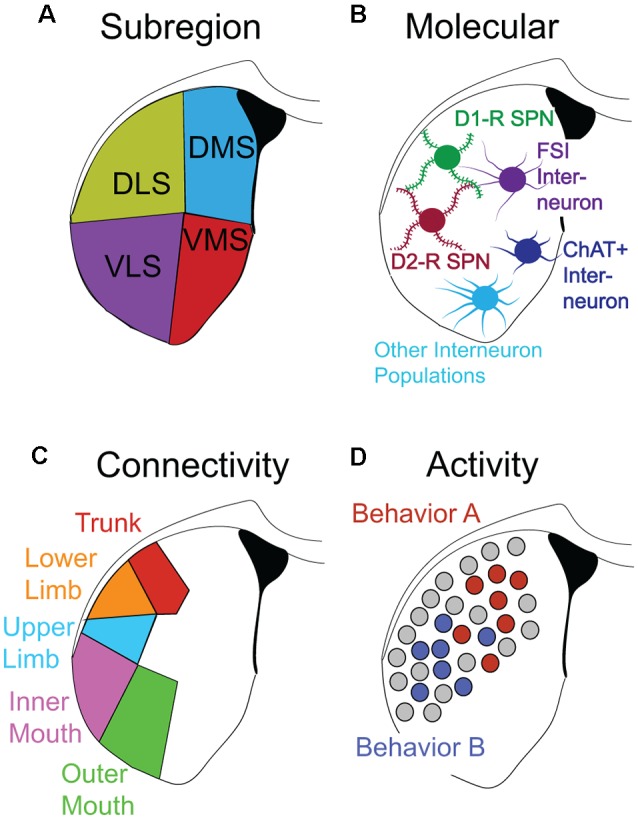
Functional definitions of striatal neurons. **(A–D)** Different dimensions/layers/’masks’ describing striatal neurons. **(A)** Striatal subregion. **(B)** Molecular/genetic: principal striatal cell types include Drd1+ SPNs, Drd2+ SPNs, PV+ FSIs, ChAT+ cholinergic interneurons, and several other important subtypes of interneuron populations. **(C)** Homuncular: striatal cells preferentially receive inputs from different regions of cortex. Sensorimotor inputs corresponding to specific body parts map to specific regions of the striatum adapted from Robbe (2018). **(D)** Task-specific recruitment: segregated clusters of neurons recruited by specific behavioral sequences (Behavior A vs. Behavior B) are shown.

A compelling hypothesis is that the long-range input/output connectivity (and local circuit structure) of a cluster of striatal neurons defines its recruitment to encoding a given S-R behavioral association (e.g., associating an auditory cue with a lever press response). Recently, it has been appreciated that unique patterns of dSPN and iSPN activity in locally concentrated clusters of SPNs correlate with the performance of specific actions (Barbera et al., 2016; Klaus et al., 2017; Markowitz et al., 2018), and that individual DLS neurons exhibit sensorimotor-relevant activity during habit performance (Rueda-orozco and Robbe, 2015). It is already known that different subregions of striatum are organized in overlapping topographic domains according to cortical input (Beckstead, 1979; Berendse et al., 1979; Hintiryan et al., 2016; Hunnicutt et al., 2016). Thus, there are multiple different dimensions along which striatal cells can be classified (depicted as dimensions, layers or “masks,” in Figure 2). One can define a striatal cell by its spatial location (Figure 2A), its neurotransmitter/cell-type identity (Figure 2B), its connectivity (Figure 2C) or its behavioral association (Figure 2D). The intersection of these dimensions is expected to define striatal ensembles encoding specific actions. Thus, a putative requirement for creating and strengthening a given behavioral S-R association might be the strengthening of specific connections between cortical neurons responsible for the representation of specific sensory inputs, and action-relevant cells in the striatum. The somatosensory organization of the striatum, which has recently been highlighted (Robbe, 2018), suggests that different actions utilize topographically dispersed ensembles of striatal neurons. Yet, these different ensembles very likely use common rules of local circuit organization and plasticity (Bamford et al., 2018; Bariselli et al., 2019) as dictated by the relatively uniform cell-type composition of the striatum.

To comprehensively map the exact circuits encoding a given specific S-R association, implementation of large-scale mapping of immediate-early gene (IEG) expression (using FISH and single-cell RNA-seq) will be invaluable. To date, many studies have examined neural activity in single brain regions, using tetrode recordings or calcium imaging, where at most hundreds of cells can be monitored. The unbiased identification of neuronal activity in basal-ganglia relevant neuronal populations and their genetic identity will be accelerated with scRNAseq, smFISH, and similar molecular techniques, followed by approaches using targeted recording of neuronal activity in defined neuronal populations (Jun et al., 2017). Such experiments will facilitate progress in localizing a specific behavior within basal ganglia circuitry. It would be especially exciting to find a specific serial path of connectivity: i.e., from a distinct cortical input through the relevant subset of striatal cells and finally to a unique output in downstream brain areas.

This achievement will enable investigators to ask crucial questions about cellular and synaptic plasticity in behavioral automaticity. Since the striatum is composed of repeating microcircuit elements, common rules are likely to prevail for the encoding of diverse actions within the striatum. Some major questions are: during the encoding of a habit, compulsion, or addiction, is the activity of dSPNs or iSPNs modulated to a greater degree? Do dSPNs and iSPNs representing the same behavior sit adjacent, in the same locally concentrated cluster? If so, do they vie for control over the same behavior, or do iSPNs primarily function to inhibit competing behaviors (Tecuapetla et al., 2016; Vicente et al., 2016; Bariselli et al., 2019)?

Once the ensemble representation of a defined S-R trace has been clearly demarcated, it will accelerate the investigation into the rules governing microcircuit organization and plasticity, as has been partially achieved recently by isolating the trace of a particular auditory stimulus within the striatum (Xiong et al., 2015; Chen et al., 2019). With some notable exceptions (e.g., Gremel and Costa, 2013), most studies have primarily examined differences in circuit properties between animals that are habit-trained vs. control animals. Ideally, one would be able to target, record and manipulate specific subsets of behaviorally relevant (Figure 2D; Markowitz et al., 2018; Bariselli et al., 2019) striatal cells according to their anatomical/“humuncular” projection patterns (Figures 2A,B; Hintiryan et al., 2016; Hunnicutt et al., 2016) and compare them to adjacent (task-irrelevant) neurons in the same animal.

In order to realize this goal, one can gain genetic access to cells participating in a given S-R association, by utilizing activity-dependent, cell-specific targeting approaches such as TRAP mice (Guenthner et al., 2013; Luo et al., 2018; Figure 2D). Similarly, connectivity-based cellular targeting (Schwarz et al., 2015; Luo et al., 2018), will enable genetic access to striatal neurons that exhibit specific input/output architecture (Figure 2C). Intersectional genetic techniques will then allow the targeting of the overlap of these two dimensions, with sub-region and cell-type resolution. Adoption of these genetic techniques will permit investigators to identify cell-specific intrinsic and synaptic plasticity within the striatum induced by a particular S-R.

Next, it will be important to test the necessity of activity patterns in genetically targeted neurons for the encoding and actuating of particular behaviors. For instance, during the development of habitual cued lever-pressing, how necessary are the striatal cells active during lever-pressing for expression of this behavior? Using optogenetic and chemogenetic approaches in combination with cell-specific targeting tools, it can be tested whether the activity of a particular ensemble or synapse-type is indispensable for a given automatic behavior and whether activation of the ensemble can induce it.

Finally, a rapidly increasing body of evidence acquired from humans with genetic mutations (Hancock et al., 2018) and adverse life experiences (Corbit, 2018; Wirz et al., 2018) that are predisposing to compulsive and addictive disorders provide further opportunities for understanding the mechanisms underlying behavioral automaticity. Here, the use of CRISPR to simulate human disease in model organisms could facilitate substantial progress in modeling and potentially reversing the pathological disorders of habitual behavior. We anticipate that increased neural circuit insight into automatic behaviors will advance treatments for human disease. Recent progress in the study of drug addiction can serve as a guiding light in this regard, as recent therapeutic approaches have been developed based on the circuit-level understanding of the plasticity induced by exposure to drugs of abuse (Creed et al., 2015; Lüscher et al., 2015; Terraneo et al., 2016).

Habit formation, expression, and related disorders are among the most fundamental topics in behavioral neuroscience, and significant progress has been made in this field. We anticipate that the next decade of research into the roles of cortico-basal ganglia circuits in supporting behavioral automaticity will involve integrating innovative molecular techniques and overlaying the different anatomical and functional representations of striatal organization. Such combined high-resolution approaches will be instrumental in pinpointing specific circuits and synapses, as well as defining basic rules of microcircuit function within the vast cortico-basal ganglia circuitry driving the development and expression of habits, compulsions, and addictions.

## Author Contributions

DL, BG and AC wrote the manuscript.

## Conflict of Interest Statement

The authors declare that the research was conducted in the absence of any commercial or financial relationships that could be construed as a potential conflict of interest.
